# Clinical nutrition as part of the treatment pathway of pancreatic cancer patients: an expert consensus

**DOI:** 10.1007/s12094-021-02674-x

**Published:** 2021-08-07

**Authors:** A. Carrato, L. Cerezo, J. Feliu, T. Macarulla, E. Martín-Pérez, R. Vera, J. Álvarez, J. I. Botella-Carretero

**Affiliations:** 1 Medical Oncology Department, Ramon y Cajal University Hospital, Alcalá University, IRYCIS, CIBERONC, Pancreatic Cancer Europe, M-607, km. 9, 100, 28034 Madrid, Spain; 2 Radiation Oncology Department, La Princesa University Hospital, Madrid, Spain; 3 Medical Oncology Department, La Paz University Hospital, IdiPAZ, Cátedra UAM-AMGEN, CIBERONC, Madrid, Spain; 4 Medical Oncology Department, Vall d´Hebrón University Hospital, and Vall d´Hebrón Institute of Oncology (VHIO), Barcelona, Spain; 5 Department of Surgery, Division of Hepatobiliopancreatic Surgery, La Princesa University Hospital, IIS La Princesa, Madrid, Spain; 6 Medical Oncology Department, Complejo Hospitalario de Navarra, Navarrabiomed, IDISNA, Pamplona, Spain; 7 Endocrinology and Nutritional Department, Príncipe de Asturias University Hospital, Alcalá University, Alcalá de Henares, Madrid Spain; 8 Endocrinology and Nutritional Department, CIBER of Physiology, Obesity, and Nutrition (CIBEROBN), Ramón y Cajal University Hospital, IRyCIS, Madrid, Spain

**Keywords:** Pancreatic cancer, Nutritional screening, Medical nutrition therapy, Enteral nutrition, Parenteral nutrition, Consensus

## Abstract

**Purpose:**

Malnutrition is a common problem among pancreatic cancer (PC) patients that negatively impacts on their quality of life (QoL) and clinical outcomes. The main objective of this consensus is to address the role of Medical Nutrition Therapy (MNT) into the comprehensive therapeutic management of PC patients.

**Methods:**

A Spanish multidisciplinary group of specialists from the areas of Medical Oncology; Radiation Oncology; Endocrinology and Nutrition; and General Surgery agreed to assess the role of MNT as part of the best therapeutic management of PC patients.

**Results:**

The panel established different recommendations focused on nutritional screening and nutritional screening tools, MNT strategies according to PC status, and MNT in palliative treatment.

**Conclusions:**

There is an unmet need to integrate nutritional therapy as a crucial part of the multimodal care process in PC patients. Health authorities, health care professionals, cancer patients, and their families should be aware of the relevance of nutritional status and MNT on clinical outcomes and QoL of PC patients.

## Introduction

The annual incidence of pancreatic ductal adenocarcinoma (PC) has been increasing and, due to its poor prognosis, PC accounts for almost as many deaths as cases [1, 2]. Estimates of temporal trends for PC incidence and mortality produced by GLOBOCAN 2018 indicate a worldwide trend towards a dramatic increase of both incidence (+ 77.7% with 356,358 new cases) and mortality (+ 79.9%; 345,181 deaths) from 2018 to 2040 [3].

Pancreatic cancer remains one of the cancers with the poorest prognosis, with an overall 5-year survival rate of about 5% [4, 5]. The impact of recent improvements in both medical and surgical treatments on 5-year survival rates has been minimal [5]. An earlier diagnosis with the identification of the high-risk population and an adequate screening program could help in achieving a higher percentage of long-term survivors. Pancreatic cancer is often diagnosed late, with only approximately 20% of patients having surgically resectable tumor at the time of diagnosis [6, 7]. Three quarters of them recur after surgery and 5-year survival is approximately 27%, while those with locally advanced or metastatic cancer have a median survival ranging from 6 to 11 months [5].

Understanding the patient experience is critical in the treatment of PC. PC is known for its debilitating symptom burden and a profound negative effect on patients’ quality of life (QoL) and treatment tolerance [8, 9]. A higher pretreatment QoL has been associated with longer overall survival (OS) in PC patients [9–11].

Malnutrition is a common problem among PC patients. Approximately, 80% of patients with PC report weight loss at the time of diagnosis and over a third have lost > 10% of their body weight [12]. Two thirds of PC patients are malnourished with anorexia at the time of first consultation [13, 14]. Additionally, 70.3% of patients developed malnutrition during chemotherapy (CT) [15, 16].

Impaired nutritional status etiology is multifactorial and includes anorexia, elevated resting energy expenditure, gastric/biliary obstruction, malabsorption, treatment side effects, tumor cytokines, etc. [17]. A percentage of weight loss greater than 5% has been associated with higher surgical site infection rates and longer hospital stay [18–20]. The lower the body mass index (BMI) and the higher the weight loss, the lower the patients’ survival [21].

Identifying patients at risk of malnutrition and delivering the most appropriate and early interventions may be beneficial for patients’ outcomes and QoL [17, 22]. Nutritional assessment and nutritional intervention at diagnosis and during treatment have been recommended for all cancer patients [20, 22–24].

Nutritional risk screening results in an increased awareness about the relevance of nutritional status in PC patients and in delivering the most appropriate and early nutritional therapy [22].

According to the European Society for Clinical Nutrition and Metabolism (ESPEN) guidelines on nutrition in cancer patients, it is recommended to periodically assess the nutrient intakes, changes in body weight and BMI from cancer diagnosis, nutritional assessment, and repeat evaluation based on the stability of the clinical situation [22].

Various nutritional screening tools have been developed and validated for identifying patients at risk of malnutrition [20, 22–24]. These tools, in conjunction with other parameters, including BMI and other markers such as albumin and prealbumin, can help guide strategies to improve patient nutrition [22].

This manuscript aims to address the role of Medical Nutrition Therapy (MNT) into the comprehensive therapeutic management of PC patients.

## Methods

In October 2020, a Spanish multidisciplinary group of specialists from the areas of Medical Oncology; Radiation Oncology; General Surgery, Endocrinology and Nutrition selected by their extensive experience in managing both PC patients and MNT, from different Spanish third level hospitals, participated in two Advisory Boards with the objective to assess the role of MNT as part of the best therapeutic management of PC patients. A streamlined guidance on the key points of nutritional therapy throughout the treatment path of a PC patient was generated.

The authors developed this consensus document based on a comprehensive literature and guidelines search and on their own experience.

An initial document was drafted and reviewed by the expert panel members. Feedback was taken into consideration until the greatest level of consensus was achieved, and the final text was then validated.

During structured consensus-based decision-making, panel members voted on draft Statements and Recommendations. The extent of agreement was determined at the end of the session held on October 13, 2020 (Table 1).

**Table 1:** Classification of extent of agreement in consensus decision-making Adapted from German Association of the Scientific Medical Societies (AWMF)—Standing Guidelines Commission [25]

Level of consensus	Extent of agreement in percent
Strong consensus	> 95% of participants agree
Consensus	> 75% ≤ 95% of participants agree
Majority agreement	> 50% ≤ 75% of participants agree
No consensus	≤ 50% of participants agree

## Results

### Nutritional screening in pancreatic cancer patients

Detecting early malnutrition signs is crucial not only at the time of diagnosis, but also at all times of the treatment pathway [20, 22–24, 26].

The Global Leadership Initiative on Malnutrition (GLIM) [27] recommended a two-step strategy for assessing malnutrition. The first step is to identify subjects “at risk” of malnutrition using any of the different validated screening tools, and the second one, determining the diagnosis and grading the severity of malnutrition [27].

- Recommendation 1: nutritional screening should be performed in all PC patients, preferably by the specialist establishing the first diagnosis (strong consensus).
  Cancer patients represent a changing scenario. Malnutrition may occur at any time and will usually be progressive [20, 22–24].
- Recommendation 2: pancreatic cancer patient nutritional status needs to be considered as a dynamic reality, which depends on multiple factors, and should be reassessed periodically during the different phases of disease treatment pathway (strong consensus).

Patients are going to be treated by different Departments (Medical Oncology, Surgery, Radiation Oncology, etc.) [28, 29].

- Recommendation 3: nutrition unit, jointly with the Departments in charge of the treatment and care of PC patients, should be an active part of decision-making processes and participate in the development of the PC patient treatment protocols (strong consensus).
- Recommendation 4: the patients’ nutritional information should be discussed in the Tumor Committee, as a valuable source of information for an adequate therapeutic approach (consensus).

### Assessment of nutritional status in pancreatic cancer patients

Among the different screening tools [20, 22–24, 30] (see Table 2), the Malnutrition Universal Screening Tool (MUST) has been validated with high sensitivity and specificity for predicting postoperative morbidity [31, 32]. A higher MUST score is associated with increased morbidity and mortality in PC patients [19].

**Table 2:** Different nutritional screening tools

Recommended by the European Society for Clinical Nutrition and Metabolism (ESPEN) [22]
Subjective Global Assessment (SGA)
Malnutrition Universal Screening Tool (MUST)
Nutritional Risk Screening (NRS 2002)
Mini Nutritional Assessment (MNA) (population > 65 years)
Short Nutritional Assessment Questionnaire (SNAQ)
No recommended specifically by ESPEN [22]
Nutritional Risk Index

*ESPEN* European Society for Clinical Nutrition and Metabolism

**Fig. 1:**
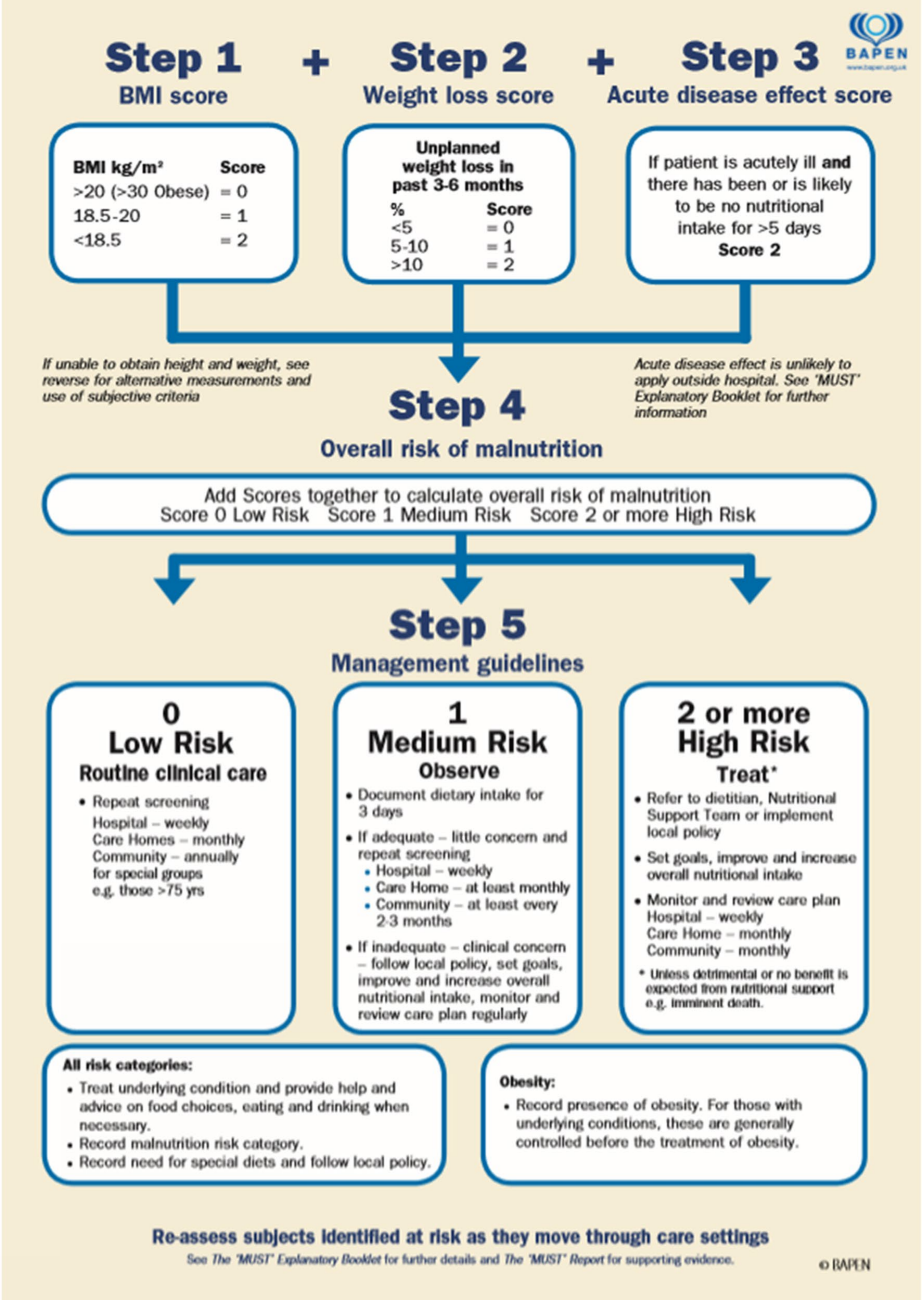
The Malnutrition Universal Screening Tool (MUST). Reproduced here with the kind permission of BAPEN (British Association for Parenteral and Enteral Nutrition). The MUST was developed by the Malnutrition Advisory Group (MAG) of BAPEN and first produced in November 2003. The MUST has been validated for use in the hospital, community and care settings, the evidence base being contained in The MUST report. An Explanatory Booklet on MUST is also available for use in training and implementation. Copies of both the Report and Booklet are available from the BAPEN Office. Reference: http://www.bapen.org.uk/pdfs/must/must_full.pdf ‘Malnutrition Universal Screening Tool’ (MUST) or weight loss chart is reproduced here with the kind permission of BAPEN (British Association for Parenteral and Enteral Nutrition). For further information on ‘MUST’ see www.bapen.org.uk’

MUST is a five-step nutritional screening tool, designed for detecting malnourished adults or those who are at risk of malnutrition [33, 34]. It can be used in hospitalized patients, community, and other care settings (Fig. 1) [33, 34].

MUST takes into account three parameters: BMI, weight loss, and the effect of acute illness. The score obtained differentiates three types of patients, namely Low risk (MUST score = 0); Medium risk (MUST score = 1); and High risk (MUST score ≥ 2) (Fig. 1) [33, 34].

- Recommendation 5: MUST should be used as screening tool in PC patients (strong consensus).
- Recommendation 6: decision-making strategy according to MUST score (strong consensus):
  o MUST = 0: control and follow-up by the Oncology or Surgery Department.
  o MUST = 1: control and nutritional treatment by the Department in charge of the patients’ treatment, starting with nutritional counseling and oral nutritional supplements (ONS). The patient must be reassessed periodically.
  o MUST ≥ 2: patient must be referred to the Nutrition Unit.
- Recommendation 7: based on MUST score, the following assessment timing is proposed (strong consensus):
  o MUST = 0 (Patient without nutritional risk): nutritional assessment every 2 months, and whenever there is any clinical change that may negatively impact on patients’ nutritional status.
  o MUST = 1 (Moderate nutritional risk patient): assess in 2–3 weeks.
  o MUST = 2 (High risk nutritional patient): assess in 5–7 days.
- In case the proposed timing cannot be met, nutritional screening should concur with the next scheduled visit.

### Nutritional medical treatments

Increased caloric requirements of patients with cancer are due to the energy requirements and biology of the tumor, the body’s reaction to the presence of the tumor, and treatments’ impact [35].

Different nutritional therapeutic strategies may be suitable in PC patients:

- *Nutritional counseling*: it is the first step and should be performed always by a health care professional [22]. It provides patients with enough knowledge about nutrition to optimize their caloric and protein intake through modifications in their alimentary behavior [22].
- *Oral nutritional supplements (ONS)*: it represents the second step. Recommended in PC patients with difficulties to achieve 100% of the intake requirements with diet alone [22]. ONS are used by 20–55% of cancer patients [36]. They should be prescribed and controlled by a health care professional. Regular follow-up is needed to monitor adverse effects and effectiveness, and make dosage adjustments, as with any medication [36].
  o Recommendation 8: specialists in charge of PC patients treatment must receive nutritional training to be able to prescribe and properly monitor ONS in PC patients. (Strong consensus).
- *Enteral nutrition (EN)*: in those PC patients unable to maintain an adequate oral intake despite counseling and ONS, supplemental or total enteral nutrition (EN) should be considered as the preferred option when gut function is preserved [22]. According to guidelines, EN may be performed using nasogastric tube or percutaneous endoscopic gastrostomy. Home EN would be usually provided through a percutaneous endoscopic gastrostomy [22, 37].
- *Parenteral nutrition (PN)*: when EN is not feasible, insufficient, or contraindicated, supplemental (SPN) or total parenteral nutrition (TPN) ensures that cancer patients receive adequate nutritional therapy [22, 37]. Prescribing, compounding, and dispensing PN is usually a multidisciplinary process which involves all members of the Nutrition Support Team (physicians, dieticians, nurses, and pharmacists) [38].

Unfortunately, grade I evidence data are still lacking to define the optimal time for initiating nutritional therapy [22]. Results from Real World Data indicate that early initiation of MNT (vs late MNT) was associated with a significant survival improvement (at least 3 months) in patients with metastatic gastrointestinal, genitourinary, and respiratory cancers [39].

### Nutrition medical treatment according to pancreatic cancer status

According to National Comprehensive Cancer Network (NCCN) guidelines, PC is classified as [40]: Resectable, Borderline resectable, Locally advanced non-resectable, and Metastatic.

This document does not intend to perform an analysis about diagnosis and decision-making process of PC treatment, but rather to introduce nutritional therapy into a patient-tailored management approach.

For nutritional purposes, we have considered three different scenarios: (1) Resectable/Borderline resectable; (2) Locally advanced; and (3) Metastatic.

#### Resectable/borderline resectable

They represent approximately 20% of PC patients [6, 7]. Although surgery is the first-choice therapy in patients with resectable PC, neoadjuvant CT and/or radiotherapy (RT) prior surgery may be considered [41]. At the time of surgery, over 70% of patients are found to have nodal metastases on pathology after resection [42, 43] and less than 7% achieved long-term cure after curative-intent surgery [41]. Resectable/borderline resectable PC patients may have occult micrometastatic disease at diagnosis.

**Fig. 2:**
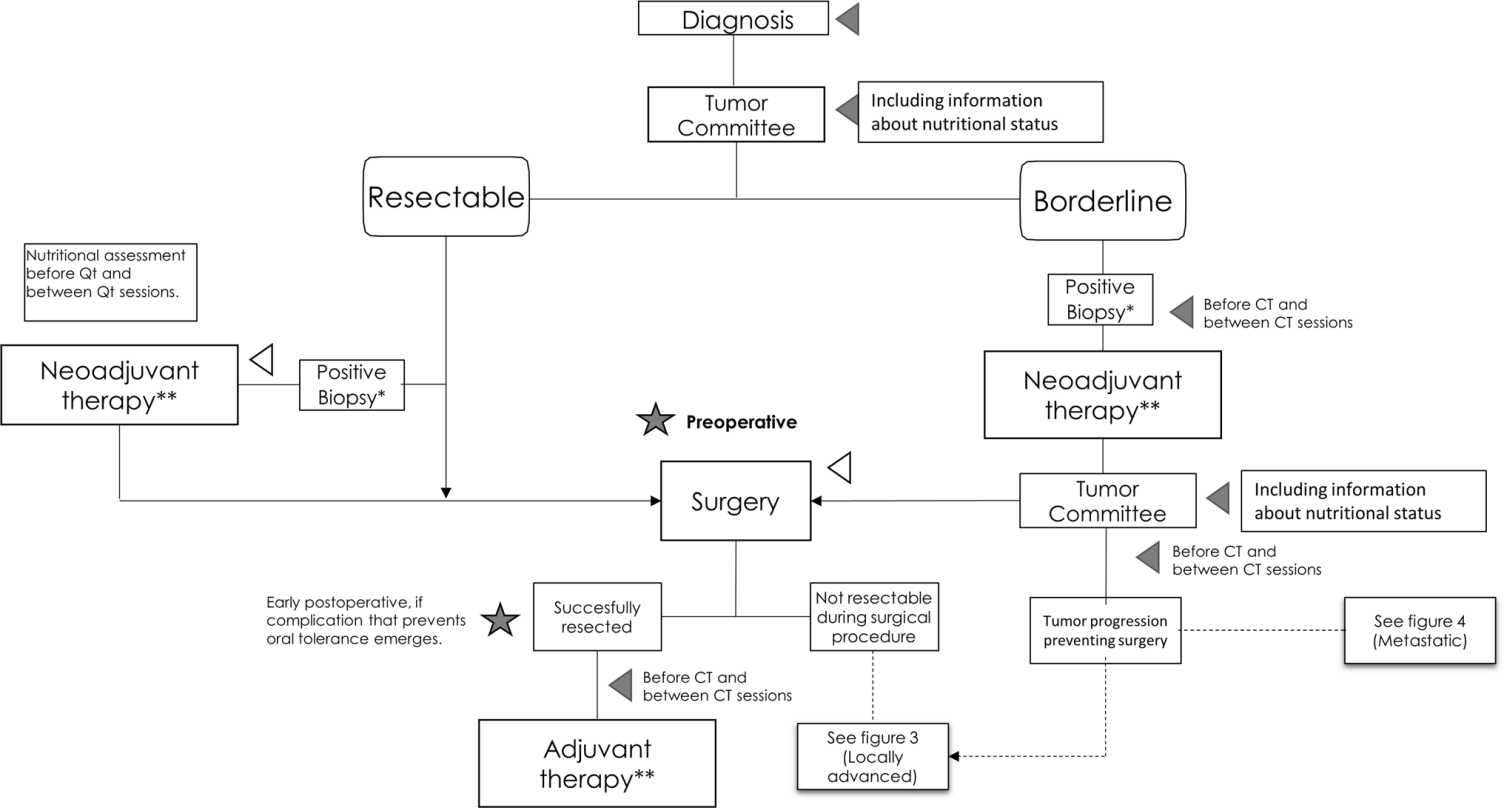
Integrating nutritional therapy within the treatment algorithm of resectable or borderline resectable pancreatic ductal adenocarcinoma. Gray triangle represents nutritional interventions and gray star represents assessment of nutritional therapy. *Fine-needle aspiration endoscopic ultrasound guidance or biopsy. **Chemotherapy ± radiation therapy or treatment as part of a clinical trial. *CT* chemotherapy

The Fig. 2 summarizes the nutritional treatment algorithm of PC resectable or borderline resectable.

Different nutritional therapeutic strategies are recommended:

A. Neoadjuvant therapy

- Recommendation 9: before starting antineoplastic treatment (CT ± RT) and between treatment sessions, perform a nutritional screening (strong consensus).
- Recommendation 10: since malnutrition symptoms may intensify during treatment, special attention should be paid to patients undergoing CT, RT, or both (strong consensus).
- Recommendation 11: prophylactic gastrostomy or jejunostomy or use of a nasogastric tube is not recommended in patients undergoing neoadjuvant treatment, although each case should be individualized according to the severity of the patient's nutritional status and their ability to a sufficient oral intake (consensus).
- Recommendation 12: there is a need to treat symptoms that may significantly impact on the patients’ nutritional status, such as anorexia, pain, malabsorption syndrome, and exocrine pancreatic insufficiency (strong consensus).

B. Surgery

a. Preoperative

- Recommendation 13: preoperative nutritional screening must be performed (strong consensus).
- Recommendation 14: in patients at risk of malnutrition, ONS should be administered for at least 5–7 days before surgery (consensus).
- Recommendation 15: in patients with severe malnutrition, delaying surgical procedure for 7–14 days should be considered. In those unable to effectively use the oral/enteral route, initiation of total or supplemental PN may be considered to improve their nutritional status (strong consensus).
- Recommendation 16: patients should be included in an Enhance Recovery After Surgery (ERAS) protocol (strong consensus).
  o ERAS protocol includes reduction of preoperative fasting period (6 h for solids and 2 h for liquids) and oral carbohydrate loading 2 h before the intervention in non-diabetic patients [44, 45].

b. Postoperative

- Recommendation 17: prolonged use of nasogastric tube should be avoided (strong consensus).
  o It is recommended to remove the nasogastric tube in the operating room.
- Recommendation 18: early onset of oral tolerance and, whenever possible, the use of postoperative oral/enteral nutrition (strong consensus).
- Recommendation 19: in case of postoperative complications that hamper oral/enteral nutrition and does not allow to cover patient nutritional requirements, early PN should be administered (strong consensus).
- Recommendation 20: if feasible, all-in-one multichamber PN bags should be used as they may reduce risk of infection and shorten preparation time at the hospital Pharmacy (consensus or majority agreement).
  o To convert multichamber bags into complete ready-to-use all-in-one admixtures, pharmaceutical assistance with expertise in compatibility and stability is mandatory. The incorporation of additional components into PN admixtures (pharmaconutrients, drugs) must be performed at the Pharmacy Service. Additionally, multichamber bags do not avoid the use of individualized all-in-one PN compounding in some patients if needed.

#### Locally advanced

Approximately 30% of patients with PC present with locally advanced disease [46, 47].

Systemic CT is considered the standard of care [48], keeping RT for some patients who do not develop distant metastases after several months of CT [49]. Although combined CT has shown a substantially improved survival, it is associated with many side effects leading to dose reduction, delays, or therapy discontinuation [50]. It is crucial to assess the nutritional status in these patients to limit its impact on increased side effects and treatment discontinuation [47, 50].

Treatment and tumors’ biology itself contribute to patients clinical and nutritional status modifications that could strongly affect the therapeutic strategy tolerance and, consequently, the OS [50, 51].

**Fig. 3:**
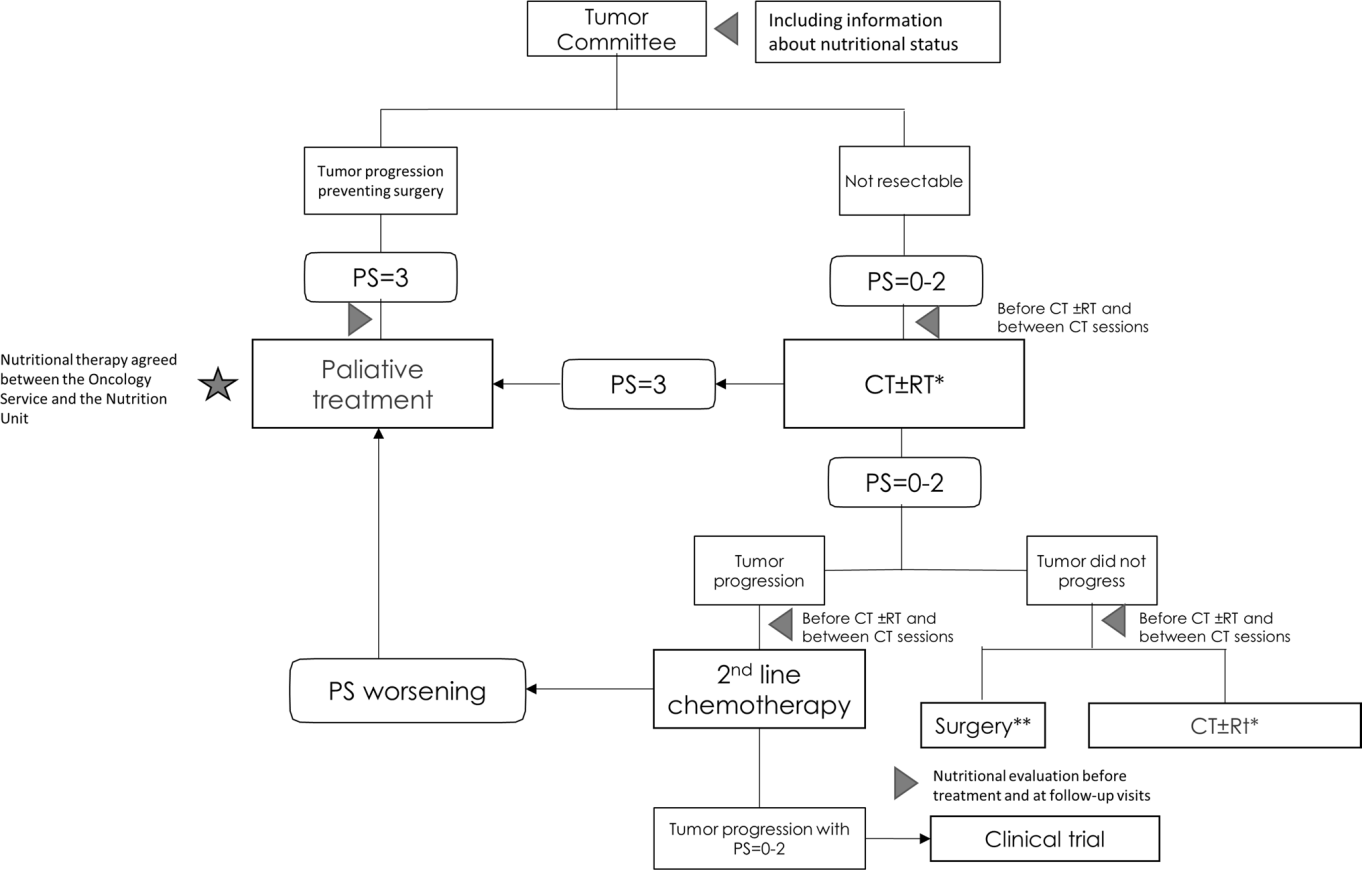
Integrating nutritional therapy within the treatment algorithm of the locally advanced pancreatic ductal adenocarcinoma. Gray triangle represents nutritional interventions and gray star represents assessment of nutritional therapy. *QT ± RT or treatment as part of a clinical trial. **See recommendations for nutritional intervention in surgery. *CT* chemotherapy, *RT* radiotherapy, *PS* performance status

Different steps are shown to establish nutritional assessment or MNT in locally advanced PC patients (Fig. 3).

For patients included in this group, recommendations from 9 to 12 and 21 are indicated.

#### Metastatic

Approximately 50% of PC patients present with metastatic disease at diagnosis [46, 47]. Metastatic PC is one of the most aggressive and highly lethal malignancies, with an estimated 5-year survival of less than 7%.

More than 80% of patients with metastatic PC suffer from significant weight loss at diagnosis [52] and over time develop cachexia [53], recognized as a major cause of reduced QoL, decreased survival, and treatment failure in patients with PC [54].

**Fig. 4:**
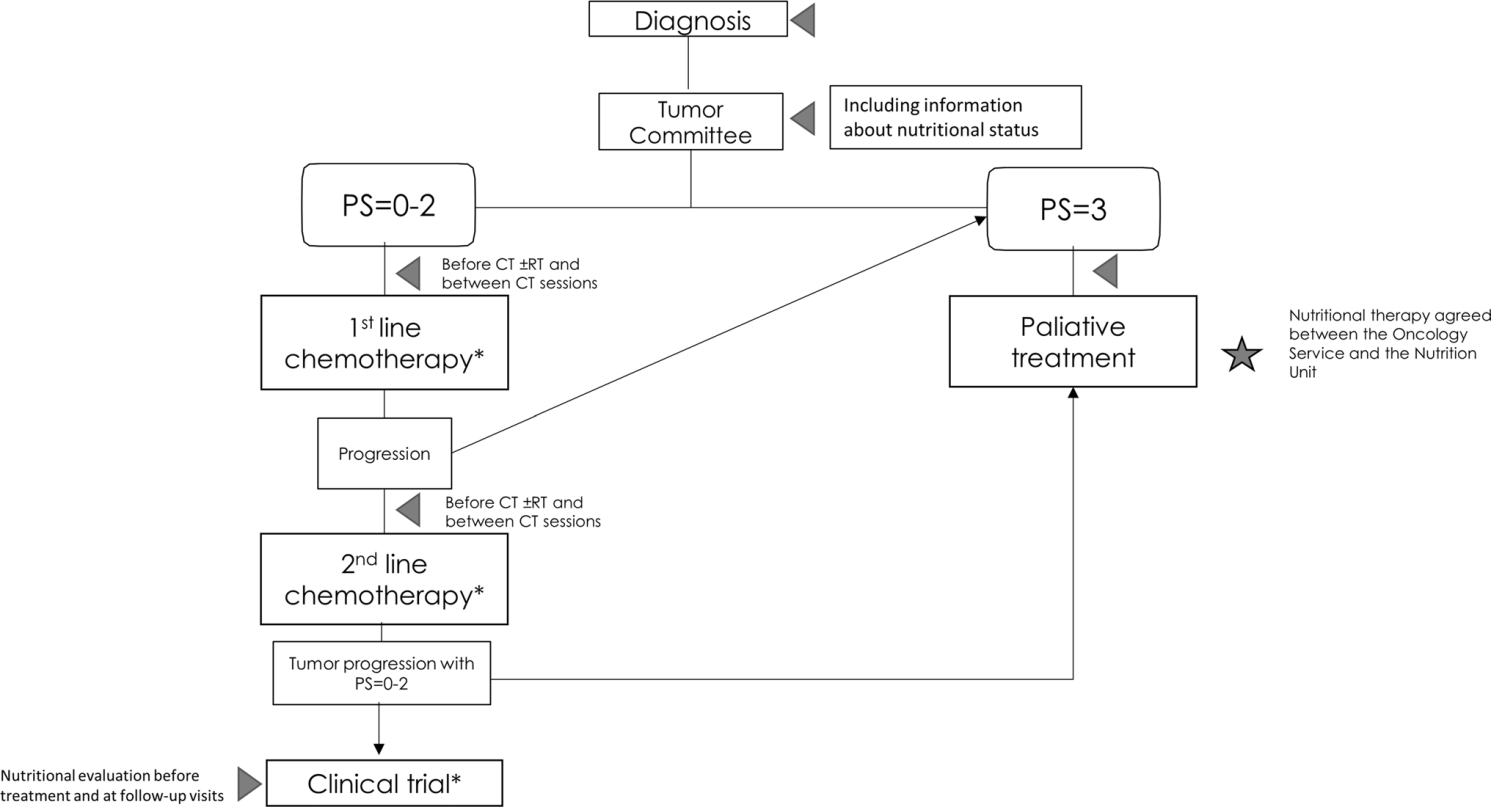
Integrating nutritional therapy within the treatment algorithm of the metastatic pancreatic ductal adenocarcinoma. Gray triangle represents nutritional interventions and gray star represents assessment of nutritional therapy. *QT ± RT or treatment as part of a clinical trial. *PS* performance status

Figure 4 shows the role of MNT as a part of the comprehensive approach to metastatic PC patient management.

For patients included in this group, recommendation from 9 to 12 are also indicated.

- Recommendation 21: nutritional therapy needs to be individualized to the patients’ clinical scenario, including performance status (PS) and prognosis. Patients’ MNT must include the participation of the Nutritional Support Team (strong consensus).

### Nutrition therapy in palliative treatment and supportive care

Pancreatic cancer should be perceived as a continuum [5, 6]. To make sound nutritional treatment decisions, it is important to understand patients and family/caregivers personal values and expectations about palliative treatment and to assess patients QoL [55, 56].

There is not unanimous agreement in the literature mainly due to the imprecise use of the term palliative care/treatment in medicine [57]. In patients undergoing palliative treatment, covering and determining their nutritional needs should be of greater importance [58]. The provided nutritional support has to be tailored to the patients’ needs, enhancing patients comfort and QoL [59, 60]. Palliative treatment should be initiated alongside standard medical care for cancer patients with dismal prognosis [61].

MNT may be integrated into a palliative treatment strategy if there are expectations of QoL improvement and there is a greater risk of dying from starvation than from disease progression [22, 62].

In patients with functioning gastrointestinal tract and insufficient oral nutrition despite nutritional counseling or ONS, EN is the preferred option [22]. When EN is contraindicated or unfeasible, PN should be recommended [22]. It is frequently used in palliative treatment for patients with head and neck, or upper gastrointestinal cancers [63]. PN should be offered and implemented considering the expected benefit on CT tolerance and consequently the potential benefit on survival [64].

- Recommendation 22: in metastatic PC patients, undergoing palliative care, a patient-tailored MNT could be implemented, with the assessment of the medical and nutritional support team (strong consensus).

ESPEN guidelines recommend home MNT, either EN or PN, in cancer patients who cannot achieve an adequate oral intake [22]. EN and PN should not be perceived as competitors, as they have clear indications and contraindications [65, 66]. Advanced-stage cancer is associated with different symptoms, including nausea, vomiting, diarrhea, abdominal pain, constipation, and gastrointestinal obstructions, therefore EN may not be a suitable option in these patients [67].

The question of whether patients with incurable cancers should receive home PN remains controversial [68]. From an ethical point of view, the debate about the convenience of feeding palliative cancer patient is still open [69]. Prognosis is a key point. According to ESPEN guidelines, home PN should be considered whether cancer patient life expectancy is greater than 2–3 months [70]. Although predicting survival in an end-stage cancer patient is anything but easy, validated scoring systems should be used [71].

- Recommendation 23: in patients with locally advanced or metastatic PC with a life expectancy greater than 3 months who present intestinal obstruction or pseudo-obstruction and decision of starting with PN to improve QoL has been adopted, home PN may be prescribed (consensus).
  o Life expectancy, ethical, legal, and logistics aspects must be taken into consideration before recommending home artificial nutrition.

## Discussion

In Europe, the incidence and mortality of PC is rising [1–3]. The advanced stage of PC at diagnosis, makes mortality rates very high despite therapy improvements [5, 72]. There is an unmet need for developing effective cancer screening tools and programs [72].

Treatment options available include surgery, RT, CT, and immunotherapy. Although immunotherapy has shown promising results in different malignancies, it has not provided positive effects in clinical trials. Only in PC patients with positive microsatellite instability, which may induce stronger anti-tumor immunity, immunotherapy might play role [73, 74].

Consequences of malnutrition in the outcomes of surgery, especially when a cephalic pancreatectomy is performed, are relevant.

Chemotherapy treatments are associated to a variety of nutrition-related symptoms such as loss of appetite, nausea, vomiting, and taste changes [75, 76], all of which interfere with the patients’ ability to eat and enjoy meals, leading to impaired nutritional intake, deterioration of the nutritional status, and decreased QoL [75, 76].

One of the earliest PC symptoms, which may precede the diagnosis by months, is unintentional weight loss [50] which leads to progressive functional impairment, increases treatment toxicities, lowers the dose intensity of CT, and impacts negatively on survival and QoL [77, 78].

Cancer cachexia is a complex and multifactorial syndrome characterized by a continuous decline in skeletal muscle mass, with or without fat loss [77] (Table 3). Its treatment should follow a patient-tailored and comprehensive approach for achieving an overall improvement of patient PS to increase QoL and tolerance of anti-tumor therapies [77, 78].

**Table 3:** Overview of different definitions of cancer cachexia Adapted from Mitchell et al. [35] and Vanhoutte et al. [78]

Study	Criteria
Evans et al. [79]	Weight loss of at least 5% in 12 months or less in the presence of underlying illness, plus three of the following criteria: Decreased muscle strength (lowest tertile) Fatigue Anorexia Low fat-free mass index Abnormal biochemistry Increased inflammatory markers (CRP > 5.0 mg/l, IL-6 > 4.0 pg/mL) Anemia (HGB < 12 g/dL) Low serum albumin (Alb < 3.2 g/dL)
Bozzetti and Mariani [SCRINIO] [80]	Weight loss ≥ 10% and presence of at least 1 symptom of anorexia, fatigue, or early satiation
Fearon et al. [EPCRC] [81]	Weight loss > 5% over past 6 months (in absence of simple starvation) or BMI < 20 and any degree of weight loss > 2% or appendicular skeletal muscle index consistent with sarcopenia (male < 7.26 kg/m^2^; female < 5.45 kg/m^2^) and any degree of weight loss > 2%

*CRP* C-reactive protein, *IL-6* interleukin-6, *HGB* hemoglobin

Drug therapy to stimulate appetite may lead to increased food intake, weight gain, and an improved QoL [82–84]. Nutritional assessment and counseling, including ONS, for advanced PC patients who are not able to achieve intake requirements is the first recommendation [35, 50, 60, 85–87]. It is crucial to improve the energy and protein content of the cachexic patient diet, who generally does not reach the requirements to maintain the PS [35, 88].

Nutritional counseling, ONS, or both were able to improve QoL, body weight, reduction of treatment adverse events, and some clinical outcomes, but not survival, in malnourished or at risk of malnutrition cancer patients receiving both CT and RT as adjuvant or neoadjuvant treatment [84, 89, 90].

Patients with exocrine pancreatic insufficiency, regardless the cause, may benefit from the administration of pancreatic enzyme supplements. There is evidence supporting the role of pancreatic enzyme supplementation in patients with exocrine pancreatic insufficiency due to chronic pancreatitis or pancreatic surgery [91, 92].

ESPEN guidelines recommend total or supplemental EN for those cancer patients who cannot maintain an adequate oral nutrition (despite counseling or ONS) [22].

In patients with obstructing head and neck cancers undergoing CT or RT, and preferably at early stages, EN provides better clinical outcomes than oral feeding [93, 94]. It was associated with reduced CT-related adverse events in patients with esophageal cancer undergoing neoadjuvant CT [95]. The results of a randomized clinical trial, conducted on malnourished upper gastrointestinal cancer patients, show that home EN provided a higher percentage of completing the planned CT regimens compared with those receiving nutritional counseling only [96].

Parenteral nutrition can be the preferred option when enteral route is insufficient or not feasible. One of the main purposes of adding PN to ONS or EN is to improve tolerance to an intensive oncology treatment by improving patients’ nutritional status [97]. A small study where PC patients received home PN (HPN), suggested that a timely onset of PN with sufficient calories leads to an improved nutritional status [98]. In any case, its use depends on patients’ characteristics and needs [22].

Total PN may be a valuable option in cancer patients with an acceptable PS and a life expectancy > 2 months, undergoing a total macronutrient deprivation [99]. Moreover, in severely malnourished aphagic cancer patients, total PN significantly increased the survival length as compared with simple hydration, even in those patients with low to moderate survival values [100].

Finally, a customized nutritional therapy program, which was escalated from counseling to ONS, EN, and PN, as required, to avoid a caloric deficit was associated with reduced hospital stay and body weight and QoL improvement [101].

Malnutrition has been documented to be an independent risk factor for surgical results, thus identifying patients at risk prior to surgery is critical to improve outcomes [102, 103].

In order to minimize the negative impact of malnutrition, PC patients should undergo early nutritional screening and nutritional therapy for optimizing clinical outcomes. Early nutritional intervention (screening and therapy) has been shown to reduce morbidity, length of stay, and admission costs in hospitalized patients [104, 105].

There are some limitations in this article. The first one is its concept. This document was built as a narrative rather than a systematic review. Second, because of the lack of high-quality published scientific evidence about the role of Clinical Nutrition in PC patients, some recommendations are mainly based on panel experience, with a potential bias. Finally, some of the recommendations are subject to the logistical viability of each hospital, so they must be adapted to the reality of each center.

## Conclusions

The development of standardized nutritional strategies in PC patients is a continuous and complex process which requires the involvement of different specialties, including Nutrition unit, Medical Oncology, Radiation Oncology, and Surgery departments.

This article shows the need to integrate nutritional therapy as a crucial part of the multimodal care process in PC patients. This issue needs to be understood and assumed by health authorities, health care professionals, cancer patients, and their families and caregivers.

Due to deficiencies in training or lack of time, oncologists, surgeons and radiotherapists do not routinely perform nutritional screening to identify patients at risk of malnutrition.

It is our hope that this article will alert clinicians who treat PC patients, to effectively manage their nutritional status. We also expect that this paper increases the awareness of the relevance of nutritional status and MNT on clinical outcomes and QoL of PC patients.

## Data Availability

Not applicable.
